# Coordinated Roles of the Putative Ceramide-Conjugation Protein, Cwh43, and a Mn^2+^-Transporting, P-Type ATPase, Pmr1, in Fission Yeast

**DOI:** 10.1534/g3.119.400281

**Published:** 2019-06-14

**Authors:** Norihiko Nakazawa, Xingya Xu, Orie Arakawa, Mitsuhiro Yanagida

**Affiliations:** G0 Cell Unit, Okinawa Institute of Science and Technology Graduate University, Onna-son, Okinawa 904-0495, Japan

**Keywords:** Cwh43, fission yeast, nutrient, manganese, Pmr1

## Abstract

Genetically controlled mechanisms of cell division and quiescence are vital for responding to changes in the nutritional environment and for cell survival. Previously, we have characterized temperature-sensitive (ts) mutants of the *cwh43* gene in fission yeast, *Schizosaccharomyces pombe*, which is required for both cell proliferation and nitrogen starvation-induced G0 quiescence. Cwh43 encodes an evolutionarily conserved transmembrane protein that localizes in endoplasmic reticulum (ER). Defects in this protein fail to divide in low glucose and lose mitotic competence under nitrogen starvation, and also affect lipid metabolism. Here, we identified mutations of the *pmr1* gene, which encodes an evolutionarily conserved Ca^2+^/Mn^2+^-transporting P-type ATPase, as potent extragenic suppressors of ts mutants of the *cwh43* gene. Intriguingly, these *pmr1* mutations specifically suppressed the ts phenotype of *cwh43* mutants, among five P-type Ca^2+^- and/or Mn^2+^-ATPases reported in this organism. Cwh43 and Pmr1 co-localized in the ER. In *cwh43* mutant cells, addition of excessive manganese to culture media enhanced the severe defect in cell morphology, and caused abnormal accumulation of a cell wall component, 1, 3-β-glucan. In contrast, these abnormal phenotypes were abolished by deletion of the *pmr1*^+^ gene, as well as by removal of Mn^2+^ from the culture medium. Furthermore, nutrition-related phenotypes of *cwh43* mutant cells were rescued in the absence of Pmr1. Our findings indicate that the cellular processes regulated by Cwh43 are appropriately balanced with Pmr1-mediated Mn^2+^ transport into the ER.

Cells have the ability to respond and adapt to changes in their nutritional environments. Switching from a proliferative state to quiescence (G0 phase) and vice versa is a principal survival strategy when confronting drastic changes of nutritional availability. The fission yeast, *Schizosaccharomyces pombe* (*S. pombe*), is a suitable model organism to study these switching mechanisms, because quiescent cells can easily be induced from proliferative cells by nitrogen (NH_4_Cl) deprivation in the culture medium (Su *et al.* 1996; Yanagida 2009). Conversely, replenishment of the nitrogen source causes quiescent *S. pombe* G0 cells to restart proliferation. Mechanisms controlling this switching are assumed to be evolutionarily conserved. Taking advantage of this unicellular organism, genetic regulation of mitotic competence (MC) to restart proliferation was investigated in G0 cells (Sajiki *et al.* 2018), as well as cell-cycle regulation in proliferative cells. Currently, more than 80 “super housekeeping (SHK) genes,” which are essential for both proliferation and quiescence, have been identified (Sajiki *et al.* 2009).

We have been interested in the fission yeast *cwh43* gene, an SHK gene that encodes a conserved transmembrane protein, potentially involved in metabolism of a wide range of nutrients (Nakazawa *et al.* 2018). In addition to the loss of viability under nitrogen-starvation, *cwh43* temperature-sensitive mutants fail to divide in low glucose, suggesting that Cwh43 is required for responses to both carbon- and nitrogen-sources. Intriguingly, *cwh43* mutant cells significantly altered levels of biomarker metabolites for nutritional stresses, and over-accumulated triacylglycerols (neutral lipids). Cwh43 has been proposed to incorporate the sphingolipid, ceramide, into a lipid moiety of glycosylphosphatidylinositol (GPI)-anchored proteins (GPI-APs) in endoplasmic reticulum (ER) in budding yeast (Martin-Yken *et al.* 2001; Ghugtyal *et al.* 2007; Umemura *et al.* 2007). GPI-anchoring is an evolutionarily conserved post-translational modification, involved in various cellular functions at the plasma membrane, such as signal transduction, cell-cell interaction, cell adhesion, and host defense (Fujita and Jigami 2008). However, the physiological role of Cwh43 protein, the so called ‘ceramide remodelase’, is still largely unknown.

To address the role of this enigmatic protein, we employed genetic screens for spontaneous extragenic mutations that recover cell division of the *cwh43-G753R* mutant at the restrictive temperature (37°). We identified mutations of an evolutionarily conserved Ca^2+^/Mn^2+^-transporting, P-type ATPase, Pmr1, which has been proposed to be involved in regulating cellular Mn^2+^ levels in *S. pombe* (Rudolph *et al.* 1989; Dürr *et al.* 1998; Maeda *et al.* 2004; Culotta *et al.* 2005). We also showed that *cwh43* mutant cells are sensitive to excess manganese, but not to calcium. Manganese is a biologically relevant trace metal that is required for growth and survival of most organisms. This trace element acts as a cofactor of many metalloenzymes involved in a wide range of cellular functions, including reactive oxygen species (ROS)-scavenging, protein glycosylation, DNA and RNA biosynthesis, phospholipid biosynthesis, and the urea cycle (Keen *et al.* 2000; Reddi *et al.* 2009; Jensen and Jensen 2014). Striking suppression of abnormalities of *cwh43* mutants by *pmr1* mutations or Mn^2+^ deprivation highlights the balanced action between Cwh43 and manganese at the ER, and suggests the importance of this metal in proper metabolism of nutrients and lipids.

## Materials and Methods

### Strain constructions

*Schizosaccharomyces pombe* strains used in this study were derived from haploid wild-type strains 972 (*h*^-^) and 975 (*h*^+^). Temperature-sensitive (ts) *cwh43-G753R* and *cwh43-G300E* strains were constructed by genomic integration of these mutation sites into the wild-type strain (Nakazawa *et al.* 2018). Deletion of the *pmr1*^+^, *pmc1*^+^, *cta3*^+^, *cta4*^+^, *cta5*^+^, and *pdt1*^+^ genes was performed by replacing the entire genomic locus with the hygromycin-resistance gene in a haploid wild-type strain. Strains expressing C-terminal GFP-tagged Cwh43-WT were described previously (Nakazawa *et al.* 2018). C-terminal mCherry-tagged Pmr1 strains were made by chromosomal integration under the native promoter with the kanamycin-resistance gene.

### Suppressor screening and identification of mutations

Suppressor screening, whole-genome sequencing, and mutation identification followed procedures described previously (Xu *et al.* 2018). Briefly, *cwh43-G753R* mutant cells were plated on YPD medium and incubated at 37° for 4 days to obtain revertant colonies (frequency; ∼1x 10^−6^) that contained suppressor mutations in addition to the original *cwh43-G753R* ts mutation. Genomic DNA of 30 revertants was extracted, and two genomic DNA mixtures were prepared and each mixture contains equal amounts of genomic DNA from 15 revertants. Then these genomic DNA mixtures were subjected to whole-genome sequencing analysis using Illumina HiSeq 2000 sequencers. Mutation sites and amino acid substitutions in the *pmr1* and *pga3* genes in the obtained suppressor strains were confirmed by Sanger dideoxy sequencing.

### Growth conditions

*S. pombe* cells were cultivated in YPD (rich medium) or EMM2 (minimal medium) (Moreno *et al.* 1991) supplemented with extra MnCl_2_, MnSO_4_, or CaCl_2_ as indicated. For Mn^2+^ deprivation, MnSO_4_ (original concentration; 2.6 μM) was removed from the recipe for EMM2 medium. To prepare EMM2 medium devoid of Ca^2+^, 0.1 mM CaCl_2_ and 2.1 μM calcium pantothenate were deleted from the recipe for EMM2. For the change from normal EMM2 to Mn^2+^-deprived or extra Mn^2+^-containing media, cells cultivated in normal EMM2 at 26° were harvested by vacuum filtration, washed in the target liquid media twice on the membrane, and transferred to the new media at 26° for 24 hr. For nitrogen starvation, cells were first cultured in normal EMM2 at 26° and then transferred to nitrogen-deficient EMM2-N medium at 26° for 24 hr (Sajiki *et al.* 2009). Cell viability was calculated as a percentage of the number of colonies formed *vs.* the number of plated cells. Numbers of liquid-cultured cells were counted using a Multisizer 3 (Beckman Coulter).

### Fluorescence microscopy and live-cell analysis

Fluorescent staining of 1, 3-β-glucan was performed using aniline blue, as previously described (Okada and Ohya 2016). Lipid droplets were stained with BODIPY 493/503 (Thermo Fisher Scientific, D3922) (Meyers *et al.* 2016). Procedures for live-cell analysis were carried out using a DeltaVision Elite Microscopy System (GE Healthcare), as described previously (Nakazawa *et al.* 2016). Silicon objective lenses (UPLSAPO 100XS; NA 1.35; Olympus) were used. All-in-one microscopes, BZ9000 and BZ-X700 (Keyence, Japan), were used to obtain bright field images.

### Data availability

Illumina sequence data have been deposited in the NCBI Sequence Read Archive under BioProject ID PRJNA533914 with BioSample accessions SAMN11471144 and SAMN11471145. Strains are available upon request. Supplemental Figure S1 shows rescue of defective phenotypes in the *cwh43-G753R* mutant cells by *pga3-R161C* mutation. Supplemental material available at Figshare: https://doi.org/10.25387/g3.8259173.

## Results

### Identification of extragenic suppressor mutations of the cwh43-G753R mutant

**Figure 1:**
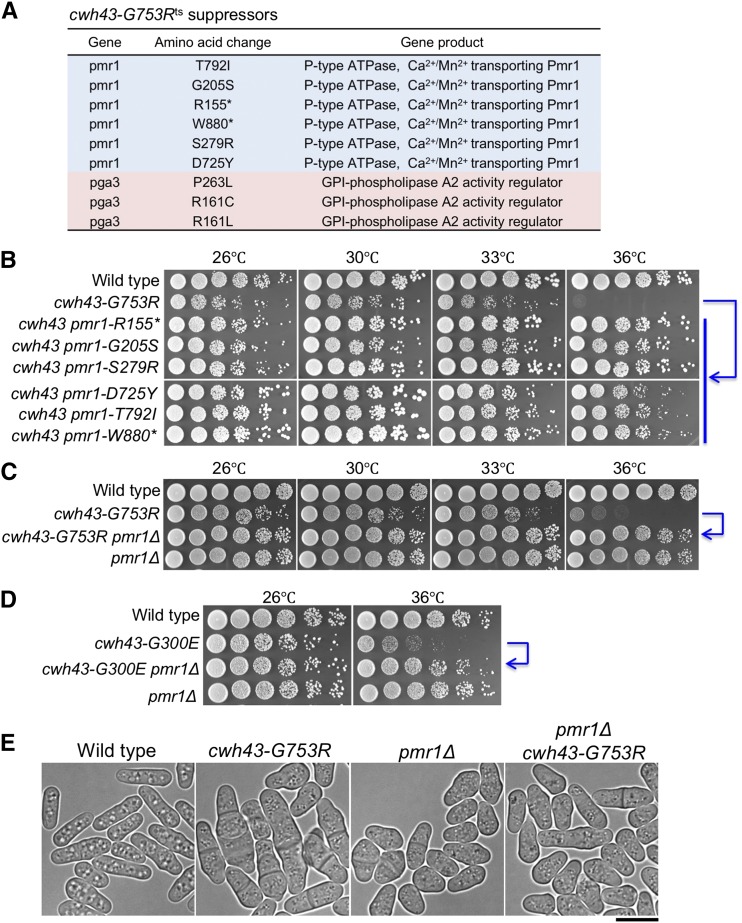
Mutations of the *pmr1* gene suppressed temperature sensitivity and morphological abnormality of *cwh43-G753R* mutant cells (A) List of isolated suppressor strains from revertant screening of the *cwh43-G753R* ts mutant. Mutated genes, amino acid changes, and gene products are shown. (B) Aliquots of WT, *cwh43-G753R*, and *cwh43-G753R* revertant strains that contain suppressor mutations were diluted serially fivefold, and were spotted onto YPD solid media at 26-36 °C. Asterisks show nonsense mutations. Blue arrows indicate rescue of temperature sensitivity. (C) WT, *cwh43-G753R*, *pmr1*Δ (deletion mutant of *pmr1*), and *cwh43-G753R pmr1*Δ double mutant strains were spotted at 26-36 °C. (D) WT, *cwh43-G300E*, *pmr1*Δ, and *cwh43-G300E pmr1*Δ double-mutant strains were spotted at 26 and 36 °C. (E) Microscopic images of WT, *cwh43-G753R*, *pmr1*Δ, and *cwh43-G753R pmr1*Δ double-mutant cells at 26 °C in bright field. Bar, 10 μm.

We previously isolated 8 alleles of *S. pombe cwh43* temperature-sensitive (ts) mutants (Nakazawa *et al.* 2018). To identify the genetic interactors of *cwh43*^+^ gene, we attempted to isolate extragenic suppressors that rescue the temperature sensitivity of the *cwh43-G753R* mutant. Among spontaneously isolated revertants of the *cwh43* mutant (frequencies; ∼10^−6^, Materials and Methods) (Xu *et al.* 2018), 30 strains were able to grow at the restrictive temperature (37°). These revertant strains were then subjected to whole genome sequencing, and suppressor mutation sites were determined in 13 strains. Three of these suppressor mutations contained missense mutations in the *pga3*^+^ gene, encoding GPI-phospholipase A_2_ activity regulator (Figure 1A). Ten of the remaining 13 suppressors contained 4 missense, 2 nonsense, and 4 nucleotide insertion or deletion mutations in the *pmr1*^+^ gene, which encodes a Ca^2+^/Mn^2+^-transporting P-type ATPase (Maeda *et al.* 2004; Cortés *et al.* 2004). Identification of *pmr1* mutations as *cwh43* mutant suppressors was unexpected, because direct involvement of Ca^2+^ or Mn^2+^ in Cwh43-mediated reactions has not been documented in *S. pombe*. Thus, we performed genetic and cytological analyses to study this suppression.

To confirm suppression of the ts phenotype in the *cwh43* mutant by *pmr1* mutations, we performed spot test analysis of these revertant strains. Although the single *cwh43-G753R* mutant failed to form colonies at the restrictive temperature (36°), all 6 *cwh43-G753R pmr1* double mutants strikingly recovered the capacity for colony formation at this temperature (Figure 1B). We further confirmed the recovery of colony formation of the *cwh43* mutant by constructing a deletion mutant of the *pmr1^+^* gene (*pmr1*Δ) and by crossing it with the *cwh43* mutant (Figure 1C). Another ts allele of the *cwh43* mutant strain, *cwh43-G300E*, was also suppressed by *pmr1*Δ, indicating that suppression of *cwh43* is not allele-specific (Figure 1D).

*S. pombe* Pmr1 is required for cell wall integrity and polarized cell growth so that the single *pmr1*Δ mutant shows small, pear-shaped cells (Maeda *et al.* 2004; Cortés *et al.* 2004) (Figure 1E). The *cwh43-G753R* mutant produces morphological abnormalities, showing elongated and swollen cell shapes (Nakazawa *et al.* 2018). However, double *cwh43-G753R pmr1*Δ mutant presented round, small cells that were indistinguishable from those of single *pmr1*Δ mutant cells. These results suggest that the ts phenotype and defective cell morphology in *cwh43* mutants depends on the presence of Pmr1.

### Deletion of the other four Ca^2+^-transporting P-type ATPases does not suppress the cwh43 mutant

**Figure 2:**
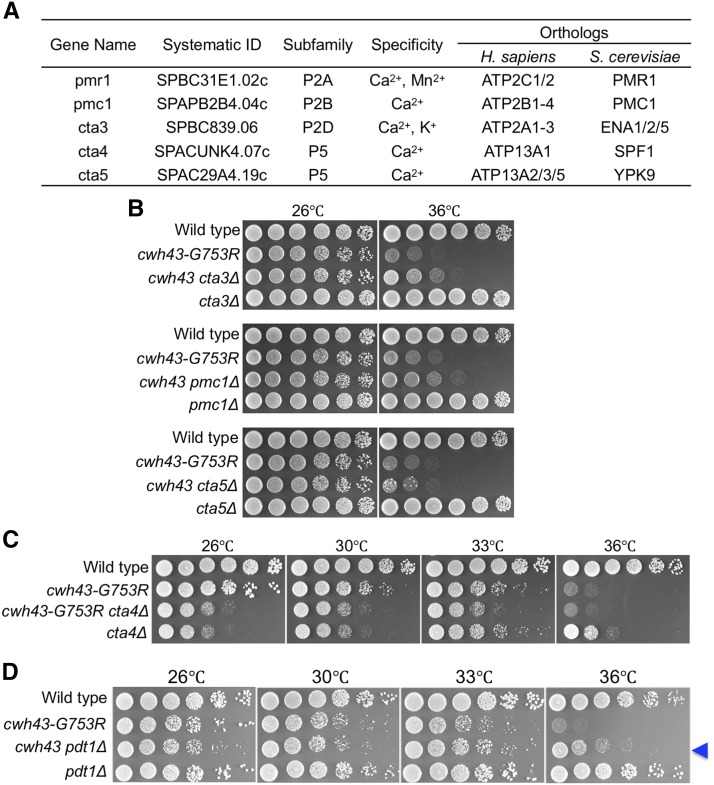
Deletion of four other Ca^2+^ P-type ATPases failed to suppress ts phenotype of *cwh43-G753R*, but the *Nramp*-related Mn^2+^-transporter, Pdt1, partly suppressed it. (A) List of 5 *S. pombe* P-type ATPases that are thought to transport Ca^2+^ and/or Mn^2+^. P-type ATPase subfamily, to which these proteins pertain, proposed ionic transport specificities in *S. pombe*, and orthologs in humans and budding yeast cells are shown. (B, C) Deletion mutants of 4 P-type ATPase genes (*cta3*, *pmc1*, *cta5*, and *cta4*) and double mutants resulting from crosses with the *cwh43-G753R* mutant were spotted onto YPD solid media at the indicated temperatures. Only the *cta4*Δ mutant strain was slightly sensitive to high and low temperatures (C). (D) Deletion mutant of the *Nramp*-related metal transporter gene, *pdt1*, was spotted along with WT, *cwh43-G753R*, and *cwh43-G753R pdt1*Δ strains. Pdt1 deletion partly rescued the ts phenotype of the *cwh43* mutant (Arrowhead).

Pmr1 has been suggested to transport Ca^2+^ in addition to Mn^2+^. Thus, we examined whether suppression of *cwh43* is caused by deletion of the other Ca^2+^-transporting P-type ATPases. The *S. pombe* genome includes 14 P-type ATPase genes, and 5 of these, including *pmr1*^+^, are thought to encode the highly conserved Ca^2+^ transporters (Figure 2A) (Ghislain *et al.* 1990; Façanha *et al.* 2002; Okorokova-Façanha *et al.* 2003; Cortés *et al.* 2004; Yoshida *et al.* 2005; Furune *et al.* 2008; Lustoza *et al.* 2011). We constructed gene deletion mutants of the remaining 4 Ca^2+^ ATPase genes, *pmc1*, *cta3*, *cta4*, and *cta5*. The resulting 4 single-deletion mutants were viable from 26° to 36°, as reported previously, although the *cta4*Δ mutant showed partial high- and low-temperature sensitivities (Figure 2BC). After crossing each mutant with the *cwh43-G753R* mutant, none of resulting 4 double-mutant strains suppressed the failure of colony formation by the *cwh43* mutation at 36°. Thus, suppression of the *cwh43* ts phenotype is specifically caused by Pmr1 deletion, but not by deletion of the other Ca^2 +^ transporters. Among the 5 Ca^2+^/Mn^2+^ P-type ATPases, thus far, only Pmr1 has been proposed to be involved in regulating cellular Mn^2+^ levels in *S. pombe* (Maeda *et al.* 2004), suggesting that loss of Mn^2^ -importing ability is critical for suppression of the *cwh43* ts phenotype.

### Nramp-related metal transporter, Pdt1, partly suppresses the ts phenotype of the cwh43 mutant

In *S. pombe*, Pmr1 regulates cell morphogenesis and Mn^2+^ homeostasis by cooperating with the evolutionarily conserved *Nramp*-related divalent metal transporter, Pdt1, which localizes at the plasma membrane (Tabuchi *et al.* 1999; Maeda *et al.* 2004). Pdt1 homologs, Smf1 and Smf2, in budding yeast, *S. cerevisiae*, take up extracellular Mn^2+^ into the cytosol with high affinity (Supek *et al.* 1996; Reddi *et al.* 2009). To test whether the ts phenotype of the *cwh43* mutant is alleviated by the loss of Pdt1, we constructed deletion mutant of *pdt1* and crossed them with the *cwh43-G753R* mutant. The resulting *cwh43 pdt1*Δ double mutant partly recovered colony formation capacity at 36°, compared to that of the *cwh43* single mutant (Figure 2D). This result suggests that the defective phenotype of the *cwh43* mutant is alleviated by restricting Mn^2+^ uptake from the extracellular environment into the cytosol.

### Cwh43 co-localizes with Pmr1 at the ER

**Figure 3:**
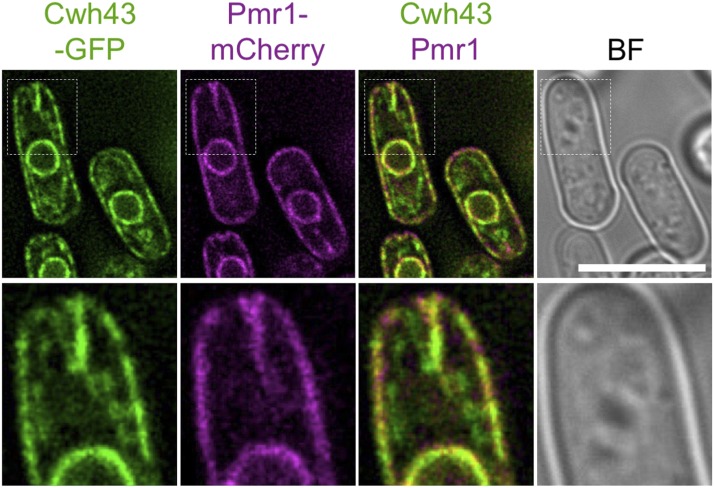
Cwh43 co-localizes with Pmr1 in close proximity to the nuclear envelope and the plasma membrane. Intracellular localization of GFP-tagged Cwh43 and mCherry-tagged Pmr1 proteins. Wild-type *cwh43*^+^ or *pmr1^+^* genes were tagged with GFP or mCherry, respectively, and integrated into the chromosome under the native promoter with the kanamycin-resistance gene. These cells were cultivated at 26°C in EMM2 media and fluorescent images were captured without fixation. A single focal plane is shown with bright field (BF) images. Inserts correspond to the white dashed boxes. Scale bar, 10 μm.

Next, we compared the intracellular localization pattern of Cwh43 with that of Pmr1. GFP-tagged Cwh43 localized at the ER, which is continuous with both nuclear and plasma membranes (Nakazawa *et al.* 2018). GFP-Cwh43 co-localized with mCherry-tagged Pmr1, which has been reported to localize predominantly at the ER in *S. pombe* (Figure 3) (Cortés *et al.* 2004), indicating that Cwh43 and Pmr1 coexists at the ER.

### cwh43 mutants are sensitive to excess manganese

**Figure 4:**
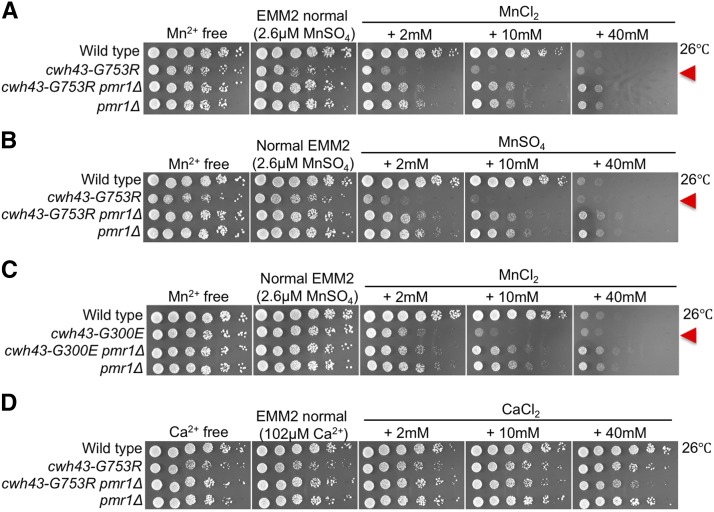
*cwh43* mutants are sensitive to excess manganese (A) WT, *cwh43-G753R*, *pmr1*Δ, and *cwh43-G753R pmr1*Δ double-mutant strains were spotted onto EMM2 minimal media, which contains 2.6 μM MnSO_4_ (normal), or onto EMM2 media containing additional manganese (2, 10, or 40 mM MnCl_2_) at 26 °C. These strains were also spotted onto manganese-free EMM2 media (Mn^2+^ free). *cwh43-G753R* is sensitive to excess manganese (arrowhead). (B) *cwh43-G753R* mutant is sensitive to excess manganese, derived from MnSO_4_. (C) *cwh43-G300E* and *cwh43-G300E pmr1*Δ strains were spotted as in (A). (D) The four strains were spotted onto EMM2 media containing 102 μM CaCl_2_ (normal), or onto EMM2 media containing additional calcium (2, 10, or 40 mM CaCl_2_) at 26 °C. These strains were also spotted onto calcium-free EMM2 media (Ca^2+^ free). Cell growth of *cwh43* mutants was sensitive to additional MnCl_2_ or MnSO_4_, but not CaCl_2_.

To test the effect of manganese on *cwh43* mutant cells, we examined cell growth of this mutant in an excess of manganese. Addition of 2 or 10 mM MnCl_2_ to normal EMM2 medium, which contains 2.6 μM MnSO_4_ as source of manganese, scarcely affected colony formation of the wild-type strain (Figure 4A). However, these manganese concentrations severely inhibited growth of *cwh43-G753R* mutant cells. The *cwh43-G753R pmr1*Δ double mutant showed mild sensitivity to the addition of 2 or 10 mM MnCl_2_, as did the *pmr1*Δ single mutant, suggesting that deletion of the *pmr1* gene partly rescues the hyper-sensitivity of *cwh43* mutant cells to excess manganese. These results were also obtained using MnSO_4_ instead of MnCl_2_, as a source of manganese (Figure 4B). The *cwh43-G300E* mutant strain also showed hyper-sensitivity to excess manganese, which was alleviated in the absence of Pmr1 (Figure 4C). The *cwh43-G753R* mutant did not show any sensitivity to excess CaCl_2_ (2-40 mM). Nor did wild type or *pmr1*Δ single-mutant cells (Figure 4D). These results indicate that cell growth in the *cwh43* mutant is probably inhibited by Pmr1-mediated manganese transport.

### The Pmr1 deletion mutant is epistatic to the cwh43-G753R mutant in cell morphology under surplus or inadequate manganese

**Figure 5:**
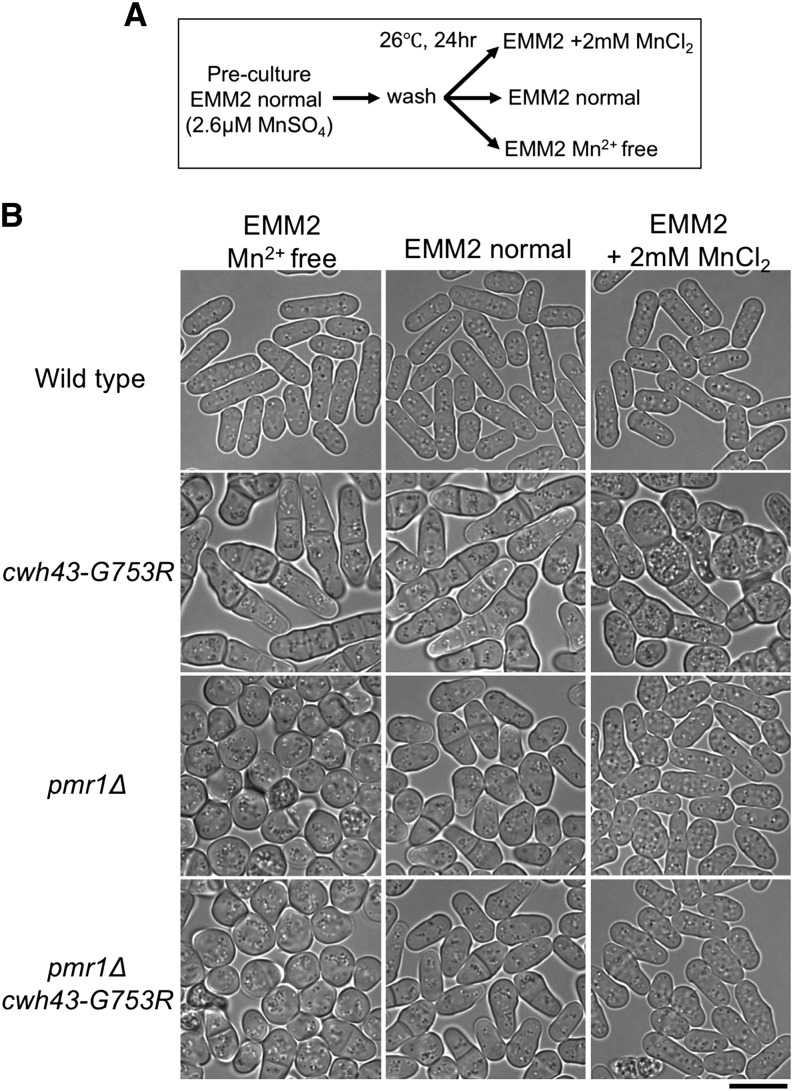
The *cwh43-G753R pmr1*Δ double mutant resembles the *pmr1*Δ single mutant in cell morphology in the presence or absence of manganese (A) Cultivation procedure. Cells were grown to mid-log phase in normal EMM2 media, and then shifted to the indicated three conditions for 24 hr at 26 °C. (B) Effect of the complete removal (Mn^2+^ free) or addition of Mn^2+^ to the medium on the morphology of WT, *cwh43-G753R*, *pmr1*Δ, and *cwh43-G753R pmr1*Δ mutant strains. Bright field images are shown. Bar, 10 μm.

Next, we examined the cell morphology of wild type, *cwh43-G753R*, *pmr1*Δ and *cwh43-G753R pmr1*Δ strains under different concentrations of manganese in the culture media. We cultivated these 4 strains in normal EMM2 liquid media that contained 2.6 μM MnSO_4_, and then shifted to 2 mM MnCl_2_-containing EMM2, Mn^2+^-free EMM2, or normal EMM2, after washing the cells with the corresponding media (Figure 5A). After cultivation for 24 hr at 26°, cell shape was not significantly affected in wild type cells in Mn^2+^-free or 2 mM MnCl_2_-EMM2 media (Figure 5B, 1^st^ row). However, in the *cwh43-G753R* mutant, abnormal cell morphology was enhanced with swollen and disorganized cell shapes in the presence of 2 mM MnCl_2_ (Figure 5B, 2^nd^ row). This aberrant morphology disappeared in the *cwh43-G753R pmr1*Δ double mutant in 2mM MnCl_2_-containing EMM2, as well as in *pmr1*Δ single-mutant cells (Figure 5B, 3^rd^ and 4^th^ rows). Instead, the *pmr1*Δ and *cwh43-G753R pmr1*Δ mutants had round cells in Mn^2+^-free EMM2, consistent with previous observations (Maeda *et al.* 2004). Taken together, the *cwh43 pmr1*Δ double mutant resembled the *pmr1*Δ single mutant in cell morphology, regardless of the presence of manganese, suggesting that deletion of *pmr1* is epistatic to the *cwh43* mutant, in terms of cell morphology.

### Manganese enhances 1, 3-β-glucan accumulation in cwh43 mutant cells

**Figure 6:**
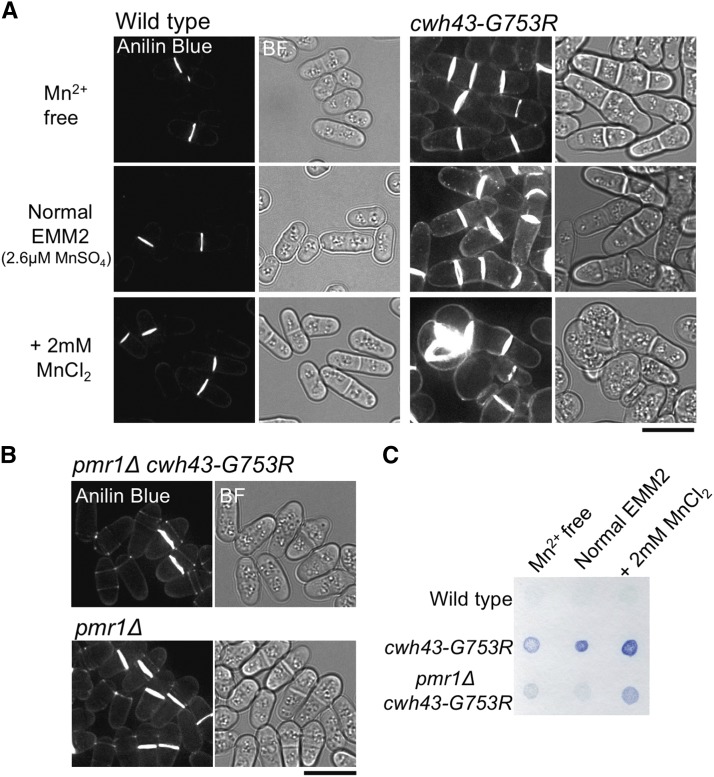
Over-accumulation of 1, 3-β-glucan in *cwh43* mutant cells was alleviated in media devoid of Mn^2+^ or in the absence of Pmr1 (A) WT and *cwh43-G753R* cells were cultured in three types of culture media at 26°C as in Figure 5, and stained for the cell wall component, 1, 3-β-glucan, using the fluorescent dye, aniline blue. Fluorescent (Aniline Blue) and bright field (BF) images are shown. Bar, 10 μm. (B) *cwh43-G753R pmr1*Δ double and *pmr1*Δ single-mutant strains were cultured in normal EMM2 medium at 26°C and stained with aniline blue as in (A). Bar, 10 μm. (C) Aniline blue-stained WT, *cwh43-G753R*, and *cwh43-G753R pmr1*Δ cells under the indicated culture conditions were spotted on filter paper.

An obvious defective phenotype of *cwh43* mutant cells is the over-accumulation of a cell wall component, 1, 3-β-glucan, which enriches at cell septa (Nakazawa *et al.* 2018). Hence, we examined whether manganese concentration in culture media affects accumulation of this glucan in *cwh43* mutant cells. Wild-type and *cwh43-G753R* mutant strains were pre-cultivated in normal EMM2 media, and then shifted to Mn^2+^-free, 2 mM MnCl_2_-containing, or normal EMM2 media. After 24 hr at 26°, intracellular localization of 1, 3-β-glucan was stained with the specific fluorescent dye, aniline blue. In wild-type cells, localization of 1, 3-β-glucan at cell septa was not obviously altered at the three Mn^2+^ concentrations (Figure 6A, left). On the other hand, in the *cwh43* mutant, the glucan signal at the cell surface was weaker in Mn^2+^-free medium than in normal or MnCl_2_-enhanced media (Figure 6A, right). Spotted cell pellets of aniline blue-stained *cwh43* mutant showed a paler blue color in Mn^2+^-free medium, compared to the two Mn^2+^-containing media (Figure 6C). These results suggest that abnormal accumulation of this glucan in Cwh43-defective cells is alleviated by manganese deprivation in the culture media.

As deletion of the *pmr1*^+^ gene partly suppressed Mn^2+^ sensitivity of *cwh43* mutants, we stained 1, 3-β-glucan in *cwh43-G753R pmr1*Δ double and *pmr1*Δ single mutants. Aniline blue staining clearly showed that over-accumulation of the glucan in *cwh43* mutant cells did not occur in the *pmr1*Δ mutant background (Figure 6B and C), indicating that glucan accumulation in defective Cwh43 is caused by Mn^2+^, which is presumably transported by Pmr1.

### Low-glucose sensitivity, loss of viability under nitrogen-starvation, and lipid accumulation in *cwh43* mutant cells were abolished in the absence of Pmr1

**Figure 7:**
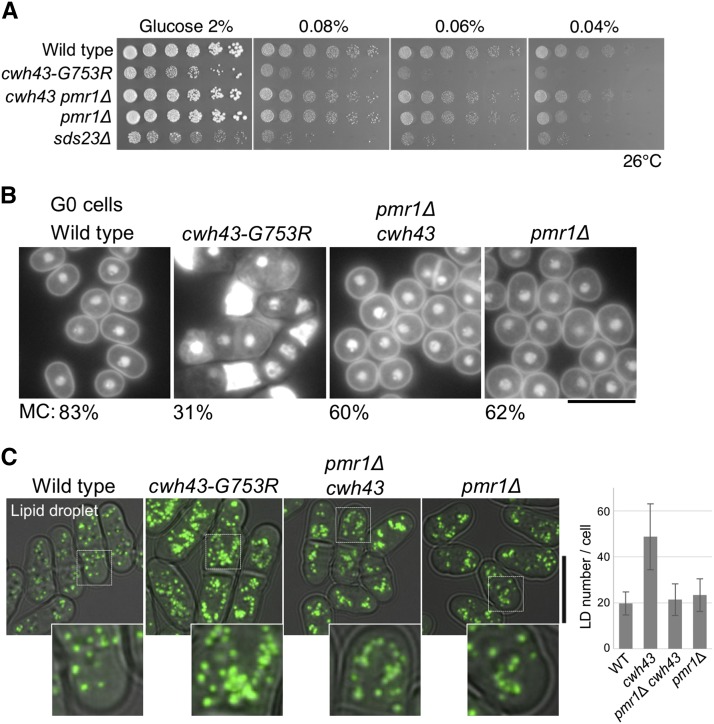
Rescue of low-glucose sensitivity, loss of mitotic competence, and lipid accumulation in *cwh43-G753R* mutant cells by Pmr1 deletion (A) Aliquots (5 × 10^4^ cells) of WT, *cwh43-G753R*, *pmr1*Δ, and *cwh43-G753R pmr1*Δ mutant strains were serially diluted (5 x), and spotted onto EMM2 media containing the indicated concentrations of glucose at 26°C. The *sds23*Δ mutant is used as a control for the low-glucose-sensitive strain (Hanyu *et al.* 2009). (B) DAPI-stained images of the indicated strains cultured after 1 day in nitrogen-deficient EMM2-N medium at 26°C. Mitotic competence (MC), which is the ability to restart cell proliferation (Sajiki *et al.* 2018), is shown. Bar, 10 μm. (C) Lipid droplets (LDs) in the four strains were stained with the fluorescent dye, BODIPY 493/503. Differential interference contrast (DIC) images were merged with fluorescence images. Inserts correspond to the area of the white dashed boxes. Bar, 10 μm. Mean ± SD of the LD number in a cell was shown (More than 50 cells were analyzed for each strain).

Characteristics of the *cwh43* mutant phenotype include sensitivity to both nitrogen starvation and glucose limitation (Nakazawa *et al.* 2018). To examine whether these *cwh43* phenotypes under nutrient deficiency are affected by deletion of Pmr1, we spotted the *cwh43-G753R pmr1*Δ double mutant on solid EMM2 media containing 0.04–2% glucose (2.2-111 mM glucose). Although the *cwh43* single mutant failed to form colonies on 0.04 and 0.06% low-glucose media, *cwh43 pmr1*Δ double mutant divided under these conditions to the same extent as wild type (Figure 7A). Under nitrogen-starvation, the *cwh43* mutant was incapable of producing spherical G0 quiescent cells like those of wild type (Su *et al.* 1996). Contrarily, the *cwh43 pmr1*Δ double mutant, as well as the *pmr1*Δ single mutant, presented spherical cells in the absence of nitrogen (Figure 7B). Mitotic competence (MC), which represents the regeneration capacity of G0 quiescent cells after nitrogen replenishment (Sajiki *et al.* 2018), diminished to 31% in the *cwh43* mutant, but was alleviated by deleting the *pmr1*^+^ gene (60%). Therefore, low-glucose sensitivity and the loss of MC in *cwh43* mutant cells require the presence of Pmr1.

We previously found that *cwh43* mutant cells overproduce triacylglycerols accompanied by lipid droplet (LD) accumulation (Nakazawa *et al.* 2018). Finally, we have verified the effect of Pmr1 deletion on lipid accumulation within *cwh43* mutant cells. Increased numbers of BODIPY 493/503-stained LDs in the *cwh43* mutant was suppressed in *cwh43 pmr1*Δ double-mutant cells (Figure. 7C). Altogether, these data suggest that Pmr1 evokes abnormal responses to nutrient deficiencies and altered lipid metabolism in Cwh43-deficient cells.

## Discussion

**Figure 8:**
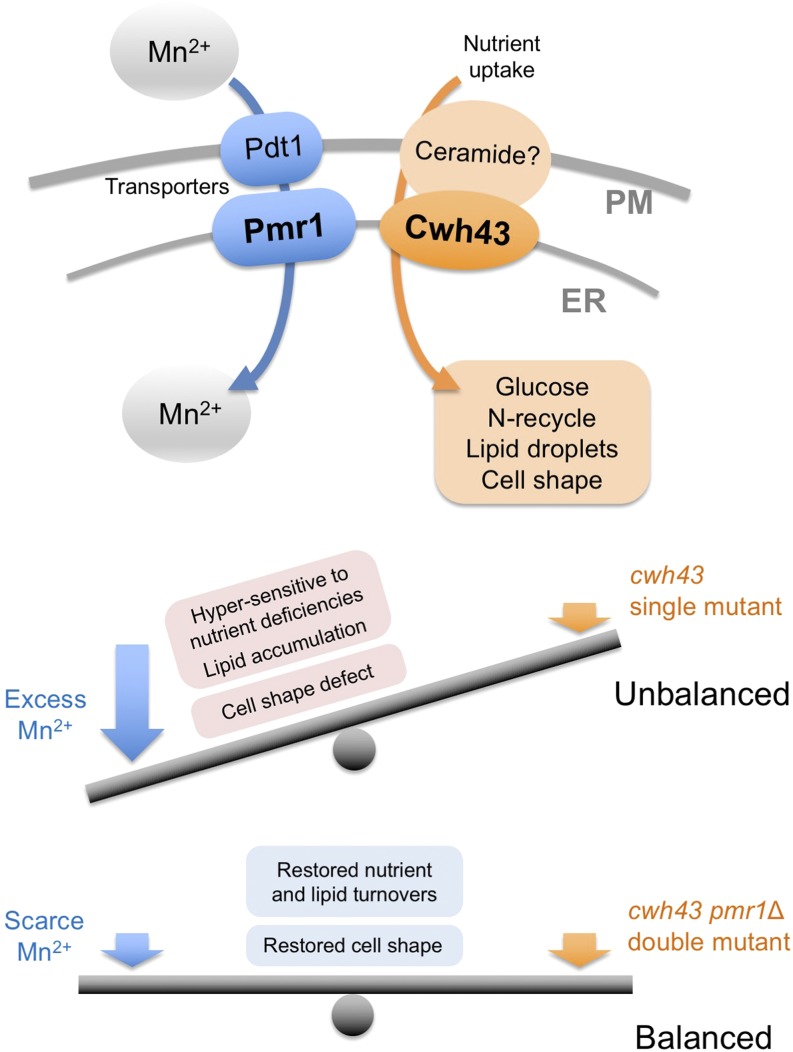
Balanced action between Cwh43 and Pmr1-mediated Mn^2+^ transport. Extracellular manganese is incorporated into cytoplasm and ER by Pdt1 and Pmr1 transporters, respectively. The putative ceramide-conjugation protein, Cwh43, is required for nutrient utilization, lipid metabolism, and cell morphology, presumably by regulating nutrient uptake into cells (Nakazawa *et al.* 2018). Defective phenotypes in *cwh43* mutants are strikingly rescued by Pmr1 deletion, indicating that the balanced action between Cwh43 and Pmr1-mediated Mn^2+^ transport at the ER controls proper nutrient and lipid turnover, as well as cell morphology. PM: plasma membrane.

Among the five reported Ca^2+^- and/or Mn^2+^-transporting P-type ATPases in *S. pombe*, we found that Pmr1 specifically recovered the defective cell growth of *cwh43* mutant cells. Our results support the idea that Pmr1 is the most relevant Mn^2+^-transporting P-type ATPase in *S. pombe*, as reported previously (Maeda *et al.* 2004; Cortés *et al.* 2004). In human cells, several P-type ATPases have been reported to facilitate Mn^2+^ uptake; however, two animal homologs of Pmr1, ATP2C1/SPCA1 and ATP2C2/SPCA2, are the only known P-type ATPases that transport Mn^2+^ into ER and Golgi with high affinity (Van Baelen *et al.* 2001; Ton *et al.* 2002; Vangheluwe *et al.* 2009; van Veen *et al.* 2014). Cortés *et al.*, (2004) and this study indicate that *S. pombe* Pmr1 localizes at the ER, closely situated to peripheral regions of the nuclear envelope and plasma membrane. Taken together, restricted incorporation of Mn^2+^ from the cytosol into the ER is likely to substantially suppress the defective phenotype of *cwh43* mutant cells (Figure 8). In other words, Cwh43 function may be indispensable for cell proliferation when Mn^2+^ is abundant at the ER.

Manganese is involved in metabolism of carbohydrates and lipids (Keen *et al.* 2000; Aschner and Aschner 2005). Recently, it was proposed that manganese stress induces cellular toxicity by affecting wide range of metabolic reactions in bacterial cells (Kaur *et al.* 2017). Considering that sensitivities to nutrient deficiencies and lipid accumulation in *cwh43* mutant are clearly rescued by Pmr1 deletion, these metabolic disorders may be caused by Pmr1-mediated Mn^2+^ transport into the ER. *cwh43* mutants present a 1000-fold reduction in the ratio of acetyl-CoA to free CoA, relative to wild-type (Nakazawa *et al.*, 2018). The acetyl-CoA/CoA ratio is believed to reflect the energy status of the cell, as does the ATP/AMP ratio (Gumaa *et al.* 1973), implying that excess Mn^2+^ in the ER perturbs nutrient metabolism for generation of high-energy compounds.

Our results raise the possibility that Cwh43 function is closely correlated with the intracellular Mn^2+^ level, particularly in the ER. Since excessive intracellular Mn^2+^ is assumed to cause cytotoxicity, regulation of this divalent cation is probably critical to manganese homeostasis. Excess intracellular manganese causes severe neurological damage, such as a Parkinson’s disease-like condition (Olanow 2004; Peres *et al.* 2016). We speculate that a potential role of Cwh43 is consumption of intracellular manganese, which is incorporated into cytoplasm and ER by Pdt1 and Pmr1, respectively. Before Cwh43-mediated ceramide conjugation, biosynthesis of GPI-anchor proteins (GPI-APs) comprises more than 20 reactions at the ER (Pittet and Conzelmann 2007; Kinoshita and Fujita 2016). GPI-AP biosynthesis includes several reactions mediated by Mn^2+^-requiring enzymes, such as glycosyltransferase (Wiggins and Munro 1998; Roseman 2001). Moreover, Pmr1 is required for protein glycosylation (Maeda *et al.* 2004; Cortés *et al.* 2004). Thus, we assume that Cwh43 is essential under conditions in which protein glycosylation is accelerated by abundant Mn^2+^ at the ER. Cwh43-mediated formation of ceramide-type GPI-APs may be linked to efficient processing of glycosylated proteins.

The *pga3* mutations were identified as *cwh43* suppressors along with *pmr1* in this study. A suppressor mutation, *pga3-R161C*, rescued colony formation at high temperature, hyper-sensitivity to excess manganese, and abnormal accumulation of 1, 3-β-glucan in the *cwh43-G753R* mutant, to a similar extent as Pmr1 deletion (Supplemental Fig. S1). Budding yeast PER1, the ortholog of *S. pombe* Pga3, is required for a precursor step of Cwh43-mediated reaction (Fujita *et al.* 2006). PER1 appeared to be involved in Mn^2+^ homeostasis through Cdc1 protein, which acts in a GPI-AP maturation step at upstream of PER1 (Paidhungat and Garrett 1998). In addition, budding yeast CWH43 and PER1 show genetic interaction with a cation-transporting P-type ATPase, SPF1 (Schuldiner *et al.* 2005; Surma *et al.* 2013; Costanzo *et al.* 2016). Although it is unclear whether the suppression of *S. pombe cwh43* mutant by *pga3* mutations occurs in a similar mechanism by the loss of Pmr1, Pga3 might thus affect the Mn^2+^ levels in the upstream process of Cwh43-mediated reaction.

Identification of Pmr1 as an extragenic suppressor of *cwh43* mutants provides an initial clue to the unexpected link between manganese homeostasis and ceramide metabolism. Ceramide has medical and dermatological importance because it blocks invasion of pathogens, allergens, and toxic compounds, and also renders the stratum corneum less susceptible to water loss (Meckfessel and Brandt 2014). Reduced ceramide abundance is correlated with atopic dermatitis (Borodzicz *et al.* 2016). On the other hand, mutations in the human Pmr1 ortholog, ATP2C1/hSPCA, cause Hailey-Hailey disease, a genetic disorder accompanied by skin blisters (Hu *et al.* 2000; Ton *et al.* 2002; Micaroni *et al.* 2016). Therefore, this potential role of ceramide metabolism in controlling manganese concentration may shed light on its cosmetic applications. Further study is required to understand the molecular mechanism underlying coordination between Cwh43 and Pmr1 in regard to manganese homeostasis.
